# Reviewed article: Borralho PM, Pavan E, Azevedo FKSF, Fontes CJF, Figueiredo AB, Brito WI, Yamanaka FR, Oliveira RG. Prevalence of human T-cell lymphotropic virus in blood donor screening at the Blood Center: a descriptive study, Mato Grosso, 2018-2021. Epidemiol Serv Saude. 2025:34;e20240799

**DOI:** 10.1590/S2237-96222026v35e20240799.a

**Published:** 2026-02-16

**Authors:** Ilka Afonso Reis

**Affiliations:** 1 Instituto Federal de Alagoas, Macéio, AL, Brazil Instituto Federal de Alagoas Macéio AL Brazil

## Peer review A

## Questionnaire

Does the manuscript contain new and significant information to justify publication? Not applicable

Does the Abstract (Summary) clearly and accurately describe the content of the article? Yes

Is the problem significant and concisely stated? Yes

Are the methods described comprehensively? No

Are the interpretations and conclusions justified by the results? Yes

Is adequate reference made to other work in the field? Not applicable

## Manuscript Structure

Length of article is: Adequate

Number of tables is: Adequate

Number of figures is: Adequate

Please state any conflict(s) of interest that you have in relation to the review of this paper. None

Interest: Average

Quality: Average

Originality: Average

Overall: Average

Recommendation: Minor Revision

## Comments

Métodos/delineamento

1) Sobre definição da “amostra”:

a) os autores colocam que o objetivo do estudo é “delinear o perfil sociodemográfico e a prevalência do HTLV-I/II entre doadores de sangue no Hemocentro de Mato Grosso entre 2018-2021”.

Sendo assim, se todas as doações no período entraram no estudo (‘’Os participantes foram todos os doadores de sangue no Hemocentro de Mato Grosso, com sede em Cuiabá, que fizeram suas doações entre janeiro de 2018 e agosto de 2021.”), na verdade, toda a população-alvo entrou no estudo e, portanto, não faz sentido falar em “amostra de conveniência não probabilística de doadores de sangue do Hemocentro de Mato Grosso”.

b) a informação a seguir deixa dúvidas sobre que amostras de sangue foram usadas no cálculo da prevalência:

“Durante os anos de 2018 a 2021, os dados foram coletados no Hemocentro de Mato Grosso, ..., onde ficam armazenadas as informações sobre todas as amostras de sangue advindas de doações voluntárias. Foram analisadas aquelas amostras retidas devido a resultados positivos no Laboratório de Sorologias, sendo o foco deste estudo, as que positivaram para HTLV-I/II.”

Que amostras de sangue participaram do estudo: todas as doações no período ou somente “aquelas amostras retidas devido a resultados positivos no Laboratório de Sorologias”?

Está realmente confuso e sugiro a reescrita dessa parte do texto para deixar claro que amostras doadas realmente fizeram parte do cálculo de prevalência (denominador dessa proporção).

2) Métodos estatísticos:

O teste qui-quadrado foi utilizado para testar que tipo de hipóteses, em que tipo de análises?

3) Resultados

a) “(...) Desses, 63 (0,10%; p = 0.02) foram reagentes para HTLV I/II.”

Esse valor-p igual a 0.02 se refere ao teste de qual hipótese sobre a prevalência?

b) A Tabela 4 seria mais informativa se trouxesse uma outra coluna com a distribuição percentual das categorias das variáveis utilizadas no grupo de amostras negativas, sem a necessidade de teste estatístico de associação, visto que esse não é o objetivo do estudo.

c) Em se confirmando que todas as amostras de sangue doadas no hemocentro durante o período de estudo participaram dele (N = 60568), ou seja, toda a população-alvo foi estudada, não há necessidade da análise estatística inferencial (teste qui-quadrado) e o estudo seria caracterizado também como descritivo. (“Estudo observacional retrospectivo e descritivo”).

4) Discussão

linha 54: “A população estudada no presente trabalho foi caracterizada por mulheres adultas.”

Essa afirmação não está correta, pois ela se baseia apenas na descrição do perfil de idade e sexo dos doadores com amostras positivas.

A população do estudo são as doações ao hemocentro no período do estudo.

Talvez a minha sugestão para incrementar a Tabela 4 ajude com essa parte.

Revisar todas as sentenças parecidas com essa na Discussão.

5) Redação

Sugiro uma revisão na redação, pois foram detectados erros básicos de concordância, uso de crase, entre outros.

